# Cognitive function has a stronger correlation with perceived age than with chronological age

**DOI:** 10.1111/ggi.13972

**Published:** 2020-07-02

**Authors:** Yumi Umeda‐Kameyama, Masashi Kameyama, Taro Kojima, Masaki Ishii, Kiwami Kidana, Mitsutaka Yakabe, Shinya Ishii, Tomohiko Urano, Sumito Ogawa, Masahiro Akishita

**Affiliations:** ^1^ Department of Geriatric Medicine, Graduate School of Medicine The University of Tokyo Tokyo Japan; ^2^ Department of Diagnostic Radiology Tokyo Metropolitan Geriatric Hospital and Institute of Gerontology Tokyo Japan; ^3^ Department of Home Care Medicine Graduate School of Medicine, The University of Tokyo Tokyo Japan; ^4^ Department of Medicine for Integrated Approach to Social Inclusion Graduate School of Biomedical and Health Sciences, Hiroshima University Hiroshima Japan; ^5^ Department of Geriatric Medicine School of Medicine, International University of Health and Welfare Narita Japan

**Keywords:** Alzheimer's disease, biomarker, face, mild cognitive impairment, perceived age

## Abstract

**Aim:**

The perceived age of older adults, as measured by their facial appearance, has been shown to be a robust biomarker of aging predictive of survival, telomere length and DNA methylation, and reportedly correlates with carotid atherosclerosis and bone status. This study aimed to determine whether metrics of dementia, including general cognition, vitality, depressive state and self‐supportability, have stronger correlations with perceived age than with chronological age.

**Methods:**

This study included 124 patients who were admitted to the Department of Geriatric Medicine, The University of Tokyo Hospital, on account of being suspected of cognitive decline. The Mini‐Mental State Examination, Vitality Index, Geriatric Depression Scale‐15, instrumental activities of daily living and Barthel Index were carried out. Five experienced geriatricians and five experienced clinical psychologists determined the perceived age of participants based on photographs.

**Results:**

The average values of the 10 raters showed excellent reliability (intraclass correlation coefficient (3, 10) = 0.941). Steiger's test revealed that perceived age showed a significantly better correlation with the Mini‐Mental State Examination (female) and Vitality Index (total, female) than did chronological age, but not with Geriatric Depression Scale‐15, instrumental activities of daily living or the Barthel Index.

**Conclusions:**

Perceived age was shown to be a reliable biomarker for cognitive assessment. **Geriatr Gerontol Int 2020; 20: 779–784**.

## Introduction

Aging is a systemic process. The most accessible organ to evaluate aging would be faces. As judged by facial appearance, the perceived age of older adults was shown to be a robust biomarker of aging that is predictive of survival, telomere length^1^ and DNA methylation.^2^ Perceived age was also reported to correlate with carotid atherosclerosis^3^ and bone status.^4^

Cognitive decline is one of the most important aspects of aging. Using chronological age and sex as covariates, Christensen *et al*. reported that the Mini‐Mental State Examination (MMSE) and cognitive score significantly correlated with perceived age.^1^ However, little beyond these findings is known about the precise relationship between cognitive status and facial appearance.

This study aimed to determine if perceived age is a better biomarker than chronological age for metrics of dementia, including general cognition, vitality, depressive state and self‐supportability.

## Methods

### *Patients*

Study participants were recruited from those who visited the Department of Geriatric Medicine, The University of Tokyo Hospital, Tokyo, Japan, presenting with forgetfulness.The following measurements were obtained: MMSE, Vitality Index,^5^ Geriatric Depression Scale‐15 (GDS‐15), instrumental activities of daily living (IADL) and the Barthel Index. We administered the same IADL scale to both male and female patients.

This study was carried out in accordance with Ethical Guidelines for Medical and Health Research Involving Human Subjects in Japan, and conformed to the Helsinki Declaration. The study protocol was approved by the institutional review board of the School of Medicine, The University of Tokyo. We provided patients and their families with detailed information regarding the study, and all participants provided written informed consent.

### *Photographs*

Participants were photographed from the front and in profile from both sides, after becoming acquainted with the participants, and their completion of a 30‐min stress check and nutritional survey.

The distance between a digital camera and participants’ faces was approximately 60 cm, and the luminous intensity was 400–600 lux.

Although middle‐aged women reportedly appear younger when wearing makeup,^6^ most participants were hospitalized and all participants did not wear makeup; however, two female participants had eyebrow tattoos.

### *Assessment of perceived age*

Five experienced geriatricians (>10 years’ experience; age range 40–60 years) and five clinical psychologists (>4 years of experience; age range 30–60 years) determined the perceived age of each participant based on the photographs.

### *Statistical analysis*

All statistical analyses were carried out with a standard spread sheet software, Excel 365, (Microsoft Corporation, Redmond, WA, USA).

The differences in the two correlation coefficients were tested with Steiger's test.^7^Correlations between perceived age and psychological tests were tested using multiple regression analysis with covariates of chronological age, sex and existence of comorbidity (hypertension, hyperlipidemia, diabetes and osteoporosis), which might influence perceived age.

## Results

### *Demographics*

A total of 129 participants joined the study. Three participants were found to be normal after investigation. Two patients were diagnosed as dementia with Lewy bodies by symptoms and imaging, which met probable dementia with Lewy bodies of McKeith criteria^8^ retroactively, two patients were diagnosed as idiopathic normal pressure hydrocephalus with images and one patient had aphasia as a result of cerebral infarction. The remaining participants were diagnosed with Alzheimer's disease (AD) based on *Diagnostic and Statistical Manual of Mental Disorders* 4th edition criteria, and their Hachinski Ischemic Scores were ≤4.^9^ Most of the patients were diagnosed using X‐ray computed tomography or nuclear magnetic resonance imaging and perfusion single‐photon emission tomography. The five non‐AD patients were excluded from the analyses; 121 AD and three normal patients were included in the analyses. No patients had paralysis or skin disease. Patient characteristics are shown in Table 1.

**Table 1:** Demographics

	Total	Male	Female	*P*
*n*	124	45	79	
Chronological age	80.9 ± 6.5	80.6 ± 6.5	81.0 ± 6.5	0.709
Perceived age	80.4 ± 3.8	81.5 ± 3.0	79.8 ± 4.1	0.0103
MMSE	21.8 ± 5.1	22.0 ± 5.0	21.7 ± 5.2	0.767
Vitality Index	9.03 ± 1.60	8.85 ± 1.64	9.13 ± 1.57	0.379
GDS‐15	5.50 ± 3.48	5.40 ± 3.19	5.56 ± 3.64	0.800
IADL	5.07 ± 2.57	4.12 ± 2.34	5.60 ± 2.53	0.00194
Barthel Index	87.9 ± 19.7	88.0 ± 21.5	87.8 ± 18.7	0.962

Data presented as the mean ± standard deviation. MMSE, Mini‐Mental State Examination; GDS‐15, Geriatric Depression Scale‐15; IADL, instrumental activities of daily living.

### *Reproducibility of the ratings*

The correlation coefficients between two raters were 0.636 ± 0.059. The intraclass correlation coefficient (ICC) was calculated as follows: the ICC(3,1) (the reliability among 10 raters) was 0.616 and the ICC(3,10) (the reliability of the average value of rates) was 0.941. These results show that although the reliability among 10 raters was moderate, the average value of the rates showed excellent reliability. Therefore, we used the average of 10 raters as the perceived age of each participant.

### *Perceived age and chronological age*

**Figure 1:**
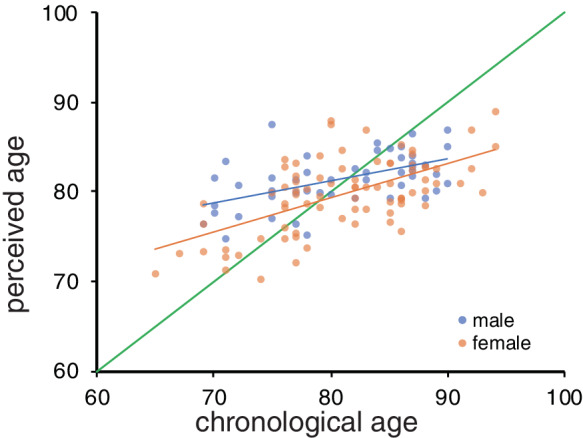
The relationship between chronological age and perceived age. The green line indicates an identical line.

A significant correlation between perceived age and chronological age was observed (*r* = 0.560, *t* = 7.47, *P* = 1.36 × 10^−11^; Fig. 1). This correlation remained significant when the analysis was limited to just men (*r* = 0.540, *t* = 4.21, *P* = 0.000127) or women (*r* = 0.606*,t* = 6.69*, P* = 3.18 × 10^−9^).

### *MMSE*

**Figure 2:**
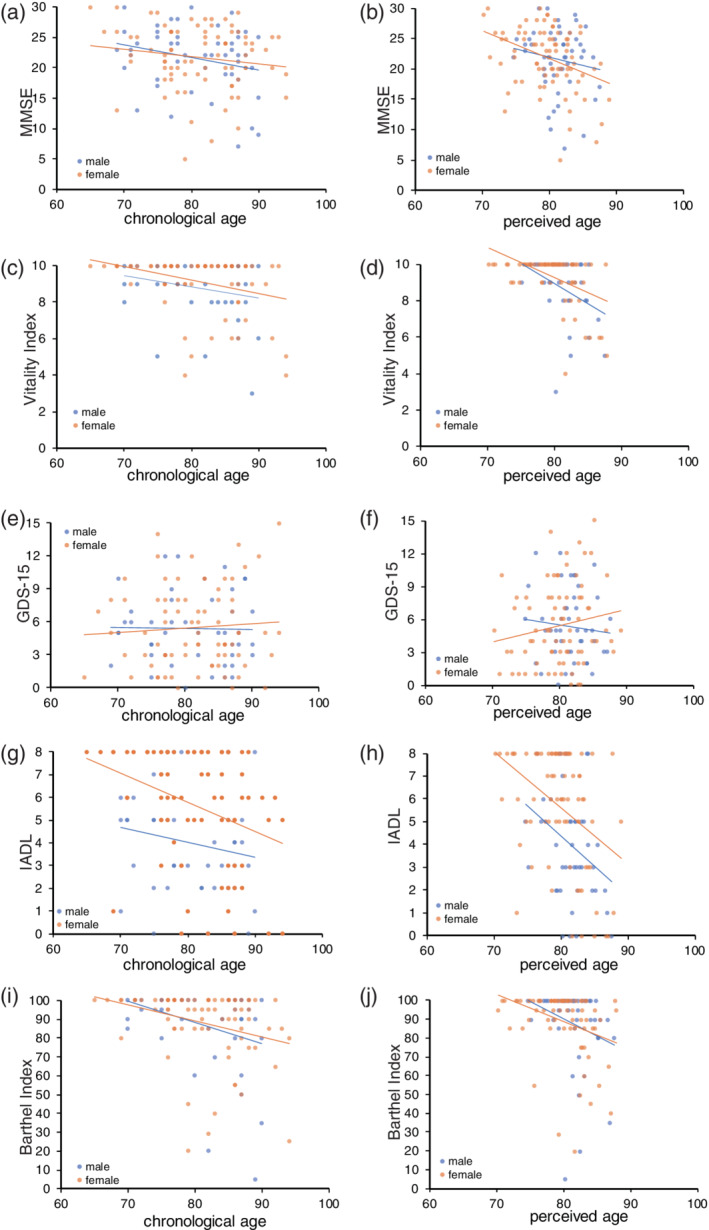
Scatter plots presenting psychological tests against chronological/perceived age. GDS15, Geriatric Depression Scale‐15; IADL, instrumental activities of daily living; MMSE, Mini‐Mental State Examination.

Scatter plots presenting the MMSE against chronological and perceived age are shown in Figure 2a and b. The MMSE was significantly correlated with both chronological and perceived age. Female perceived age was more strongly correlated with the MMSE than chronological age. Steiger's test showed significant differences in the two correlation coefficients of the female MMSE with perceived and chronological age (*P* = 0.0322; Table 2).

**Table 2:** Correlation between psychological tests and chronological/perceived age

MMSE		*r*	*t*	*P*	Vitality Index		*r*	*t*	*P*
Total	Chronological	−0.177	−1.98	0.0498*	Total	Chronological	−0.281	−3.12	0.00227*
(*n* = 123)	Perceived	−0.294	−3.39	0.000947^*^	(*n* = 116)	Perceived	−0.491	−6.01	2.27 × 10^–8^*
	Difference		1.43	0.155		Difference		2.73	0.00741*
Male	Chronological	−0.200	−2.25	0.0298*	Male	Chronological	−0.203	−2.22	0.0326*
(*n* = 45)	Perceived	−0.159	−1.78	0.0828	(*n* = 40)	Perceived	−0.345	−3.93	0.000351^*^
	Difference		0.28	0.780		Difference		0.94	0.352
Female	Chronological	−0.163	−1.82	0.0723	Female	Chronological	−0.324	−3.66	0.000472*
(*n* = 78)	Perceived	−0.370	−4.39	3.64 × 10^–5^*	(*n* = 76)	Perceived	−0.550	−7.03	8.48 × 10^–10^ *
	Difference		2.18	0.0322*		Difference		2.57	0.0122*
GDS‐15		*r*	*t*	*p*	IADL		*r*	*t*	*P*
Total	Chronological	0.044	0.48	0.632	Total	Chronological	−0.259	−2.91	0.00436*
(*n* = 118)	Perceived	0.083	0.90	0.372	(*n* = 120)	Perceived	−0.416	−4.97	2.27 × 10^–6^*
	Difference		0.44	0.657		Difference		1.96	0.0520
Male	Chronological	−0.028	−0.30	0.767	Male	Chronological	−0.139	−1.53	0.135
(*n* = 43)	Perceived	−0.115	−1.25	0.219	(*n* = 43)	Perceived	−0.342	−3.96	0.000292^*^
	Difference		0.61	0.547		Difference		1.35	0.185
Female	Chronological	0.080	0.86	0.393	female	Chronological	−0.347	−4.02	0.000138*
(*n* = 75)	Perceived	0.165	1.80	0.0762	(*n* = 77)	Perceived	−0.399	−4.73	1.04 × 10^–5^*
	Difference		0.82	0.416		Difference		0.55	0.582
Barthel Index		*r*	*t*	*P*					
Total	Chronological	−0.312	−3.60	0.000460*					
(*n* = 122)	Perceived	−0.302	−3.48	0.000709*					
	Difference		0.12	0.903					
Male	Chronological	−0.319	−3.68	0.000667*					
(*n* = 43)	Perceived	−0.242	−2.73	0.00926*					
	Difference		0.52	0.608					
Female	Chronological	−0.310	−3.57	0.000620*					
(*n* = 79)	Perceived	−0.353	−4.14	8.84 × 10^–5^*					
	Difference		0.46	0.646					

* *P <* 0.05.

*P*, probability; *r*, correlation coefficient; *t*, *t*‐value.

### *Vitality Index*

Scatter plots presenting the Vitality Index against chronological and perceived age are shown in Figure 2c and d. The Vitality Index significantly correlated with both chronological and perceived age. Perceived age was more strongly correlated with the Vitality Index than chronological age. Steiger's test showed significant differences in two correlation coefficients between total and female Vitality Index and chronological age, and between total and female Vitality Index and perceived age (*P* = 0.00741, 0.0122, respectively; Table 2). The difference in the two correlation coefficients between male Vitality Index and perceived/chronological age was not significant; considering that the scatter plot (Fig. 2c,d) did not show that the Vitality Index differed according to sex, this finding might be attributable to the small number of male patients.

### *GDS‐15*

Scatter plots presenting the GDS‐15 against chronological and perceived age are shown in Figure 2e and f. No significant correlations with GDS‐15 were found (Table 2). The difference in the two correlation coefficients between GDS‐15 and perceived/chronological age was not significant.

### *IADL*

Scatter plots presenting IADL against chronological and perceived age are shown in Figure 2g and h. IADL correlated significantly with both chronological and perceived age. Although perceived age was more strongly correlated with IADL than with chronological age, the difference between perceived age and chronological age was not significant in any group (Table 2).

### *Barthel Index*

Scatter plots presenting the Barthel Index against chronological and perceived age are shown in Figure 2i and j. The Barthel Index significantly correlated with both chronological and perceived age. The difference between perceived age and chronological age was not significant in any group (Table 2).

### *Effect of comorbidities*

Perceived age correlated significantly with MMSE, Vitality Index and IADL by multiple regression analysis excluding the effect of chronological age, sex, hypertension, hyperlipidemia, diabetes and osteoporosis (Table 3).

**Table 3:** Multiple regression analyses

	MMSE (*n* = 123)		Vitality (*n* = 116)		GDS‐15 (*n* = 118)		IADL(*n* = 120)		Barthel (*n* = 122)	
	*β*	*P*	*β*	*P*	*β*	*P*	*β*	*P*	*β*	*P*
Perceived age	−0.318	0.004955^*^	−0.503	3.874 × 10^–6*^	0.091	0.4424	−0.327	0.001854^*^	−0.209	0.05951
Chronological age	0.021	0.8484	−0.005	0.9612	−0.042	0.7240	−0.103	0.3211	−0.207	0.06245
Sex	0.086	0.3783	−0.003	0.9758	0.000	0.9990	−0.184	0.04425	0.071	0.4613
Hypertension	0.007	0.9446	0.138	0.1178	0.183	0.06795	0.175	0.04737	0.090	0.3363
Hyperlipidemia	0.084	0.4027	0.004	0.9700	−0.054	0.6083	0.020	0.8302	0.039	0.6946
Diabetes	0.082	0.4014	0.118	0.1945	−0.156	0.1321	−0.065	0.4773	−0.047	0.6240
Osteoporosis	−0.035	0.7141	−0.019	0.8309	0.047	0.6401	0.004	0.9666	0.030	0.7480

* *P <* 0.05.

*β*, standardized partial correlation coefficient; *P*, probability.

## Discussion

We have successfully demonstrated that perceived age showed a significantly better correlation with MMSE (female) and Vitality Index (total, female) than chronological age, but not with GDS‐15, IADL and Barthel Index. Furthermore, perceived age correlated significantly with MMSE, Vitality Index and IADL excluding the effect of chronological age, sex and comorbidities, which might affect perceived age. The present study thus showed that perceived age was a good biomarker of cognitive assessment that could reflect general aging, which is associated with dementia. As cognitive decline was expressed in a patient's face, a system, such as artificial intelligence, would detect the cognitive decline on a face. The present results were concordant with and elaborate on those of a previous study, which showed that the MMSE and cognitive score significantly correlated with perceived age with covariates of chronological age and sex.^1^ The present findings further support the use of facial appearance and perceived age to inform cognitive assessment. Perceived age would reflect general aging, which can cause dementia. The geriatricians feel that advanced AD patients show specific appearance, including reduction of facial expression and less attention to their appearance, which might contribute to higher estimates of perceived age. The difference in the correlation of MMSE with perceived age and chronological age was significant in female participants; this finding might be attributable to the tendency of cognitively healthy older women to pay more attention to their appearance relative to their cognitively impaired counterparts.

Although vitality was shown to be expressed on facial appearance, depression did not show a significant difference between chronological and perceived age, surprisingly. As facial expression affects perceived age, we used photographs with neutral expression in the present study.^10^, ^11^ However, even static non‐expressive faces were reported to reflect depressive states.^12^ Contrary to expectations, the present findings suggested that depression was not significantly affect perceived age. The possibility to detect cognitive decline without the disturbance of depression would be useful.

The standard deviation of perceived ages was smaller than that of chronological age. This finding may be attributable to the raters not having known the age range of the participants; specifically, the raters may not have expected patients aged in their 60s, as most of the patients in our department are aged >70 years. However, considering that linear transformation does not affect the correlation coefficients, the difference in the range would not have affected the analysis.

The present study was subject to the following limitations. First, although perceived age showed high ICC, we recruited only a minimal number of raters.^13^ Further study on facial appearance and cognitive states with more raters will be needed. Second, two female participants had eyebrow tattoos. Although middle‐aged women were reported to look younger when wearing makeup^6^, the influence of these tattoos was considered to be limited. Third, although we excluded patients with non‐AD dementia, some of our participants might have other pathologies other than AD. Fourth, most of the present patients were aged 70–90 years. Application of the conclusions to other age groups should be circumspect.

## Disclosure

KK belongs to an endowed chair funded by donations from Mr Kazuteru Noguchi, JSH, Japan Home Medical Care, Towa Pharmaceutical, Sawai Pharmaceutical, AKTIOCorporation and Ain Pharmaciez. The other authors declare no conflict of interest.
